# Empirical validation of a touchscreen probabilistic reward task in rats

**DOI:** 10.1038/s41398-020-00969-1

**Published:** 2020-08-13

**Authors:** Brian D. Kangas, Lisa M. Wooldridge, Oanh T. Luc, Jack Bergman, Diego A. Pizzagalli

**Affiliations:** 1 McLean Hospital, Belmont, MA USA; 2 Harvard Medical School, Boston, MA USA

**Keywords:** Clinical pharmacology, Learning and memory

## Abstract

Anhedonia, the loss of pleasure from previously rewarding activities, is implicated in several neuropsychiatric conditions, including major depressive disorder (MDD). In order to accelerate drug development for mood disorders, quantitative approaches are needed to objectively measure responsiveness to reward as a means to identify deficits. One such approach, the probabilistic reward task (PRT), uses visual discrimination methodology to quantify reward learning. In this computerized task, humans make visual discriminations, and probabilistic contingencies are arranged such that correct responses to one alternative are rewarded more often (rich) than correct responses to the other (lean). Healthy participants consistently develop a response bias in favor of the rich alternative. However, participants with MDD typically exhibit lower response biases, and this blunting correlates with current and future anhedonia. The present studies validated a touchscreen-based PRT in rodents with formal and functional similarity to the human task. First, rats were trained to discriminate between two lines that differed in length. Next, parametric manipulations of probabilistic contingencies, line-length stimuli, and drug treatment (amphetamine, 0.32–3.2 mg/kg; scopolamine, 0.1–1.0 mg/kg; oxycodone, 0.1–1.0 mg/kg) on response bias were evaluated. Results demonstrated orderly shifts in bias and discriminability that varied as a function of, respectively, the asymmetry of rich/lean probabilities and disparity in line lengths. Drugs that enhance reward responsiveness (amphetamine and scopolamine, but not oxycodone) increased bias, verifying pharmacological task sensitivity. Finally, performance outcomes under optimized conditions were replicated in female rats. Collectively, the touchscreen-based rodent PRT appears to have high preclinical value as a quantitative assay of reward learning.

## Introduction

Anhedonia, the loss of pleasure from previously rewarding activities, is a symptom of various neuropsychiatric conditions^1–3^, including major depressive disorder (MDD)^4^, bipolar disorder^5^, schizophrenia^6^, post-traumatic stress disorder^7^, and substance use disorders^8^. Despite its transdiagnostic importance, there are no approved medications to treat anhedonia. Critically, although positive mood restoration is currently not a diagnostic criterion for MDD^1^ or a criterion for Food and Drug Administration medication approval, MDD patients consider recovery to be a restoration of positive mood rather than a reduction in depressed mood^9^. In this regard, front-line antidepressants such as selective serotonin reuptake inhibitors are typically ineffective in restoring hedonic tone^10,11^. This is problematic because anhedonia has been found to predict unfavorable outcomes regardless of treatment modality (e.g., pharmacological, psychological, neurostimulation), disease chronicity, and increased suicide risk^12^. Thus, there is an urgent need for novel treatment strategies to restore positive mood in anhedonic patients.

Preclinical research aiming to identify novel treatment strategies for anhedonia has suffered to date from (1) a lack of precise and objective assessment tools, (2) disagreement on the very definition and conceptual boundaries of anhedonia, and (3) the use of substantially different assays across species. With respect to the first point, clinical assessment of anhedonia historically has relied on self-report questionnaires^13–15^. However, these instruments have shown poor reliability, especially in MDD, which has well-documented high heterogeneity^16^, and rarely map onto modern conceptualizations of reward processing, which have identified distinct subdomains of reward processing (e.g., Research Domain Criteria (RDoC) Positive Valence Systems^17^). With respect to the second point, although reward responsiveness alone does not reflect the multifaceted construct of anhedonia^12,16,18^, recent endeavors have focused on quantification of reward learning as a useful means of experimentally interrogating anhedonic processes. Such reappraisals have led clinical researchers to develop quantitative assays in which reward deficit profiles can be objectively characterized in laboratory settings to facilitate the identification of novel therapeutic approaches. Critically, assays of reward responsiveness in human participants that rely on behavioral outcomes also lend themselves to reverse translation for preclinical studies in nonhuman subjects and, thereby, may help address the third limitation of prior work in this area.

The probabilistic reward task (PRT^19^; modified after^20^) has been designed to provide an objective measure of *reward learning* (i.e., ability to modulate adaptive behavior as a function of reinforcement history) and is a recommended assay to probe the Positive Valence Systems in the latest revision of the RDoC matrix^21^. Based on signal detection theory^22–24^, the PRT uses visual discrimination methodology to quantify reward responsiveness and learning. In the prototypical computerized task, human participants are instructed to discriminate between two briefly presented (100 ms) mouths that vary slightly in length on a cartoon face (e.g., 13.0 mm mouth: left response key; 11.5 mm mouth: right response key) across trials. Unbeknownst to the participants, probabilistic contingencies are arranged so that correct responses on one alternative are rewarded three times more often (e.g., long line: rich alternative) than correct responses on the other alternative (e.g., short line: lean alternative). As predicted by signal detection theory, healthy control participants consistently develop a response bias in favor of the rich alternative and do so without disruption in overall task discriminability^19,25^. However, participants with anhedonia typically exhibit a lower response bias relative to healthy controls^19,26^. Critically, blunted reward learning has been repeatedly documented to correlate with current and predict future anhedonia across multiple samples^26–31^.

This apparent correspondence between decreased reward learning in the PRT and related elements of anhedonia in affective disorders has led researchers to develop animal models based on the human PRT task (*reverse translational assessment*), with the expectation that this methodology might help bridge the preclinical gap between therapeutic discovery and treatment^32^. One initial effort yielded a rodent analog of the PRT in which rats were trained to discriminate between two auditory tones that varied in duration, and a correct response on the rich alternative was rewarded three times more frequently than the lean alternative^33^. As in humans, these contingencies produced a response bias in favor of the rich alternative. Furthermore, as previously reported in human participants^34^, low doses of the direct dopamine D_2_-family agonist pramipexole (putatively through presynaptic autoreceptor activation) *decreased* response bias in rats, whereas the indirect monoaminergic agonist *d*-amphetamine *increased* response bias^33^. In addition, task sensitivity to social stress was confirmed, with rats exposed to social defeat exhibiting a blunted response bias relative to nonstressed controls^35^. The effects of social stress and drug treatment on the auditory PRT were recently replicated by Lamontagne et al.^36^, highlighting the reproducibility of such findings across laboratories. Although valuable, these early efforts are characterized by two limitations. First, although the human and rodent variants are functionally analogous, they use different stimulus modalities (humans: visual; rats: auditory). Second, training rats to criterion in the auditory PRT is labor-intensive (requiring ~40 training sessions).

**Fig. 1:**
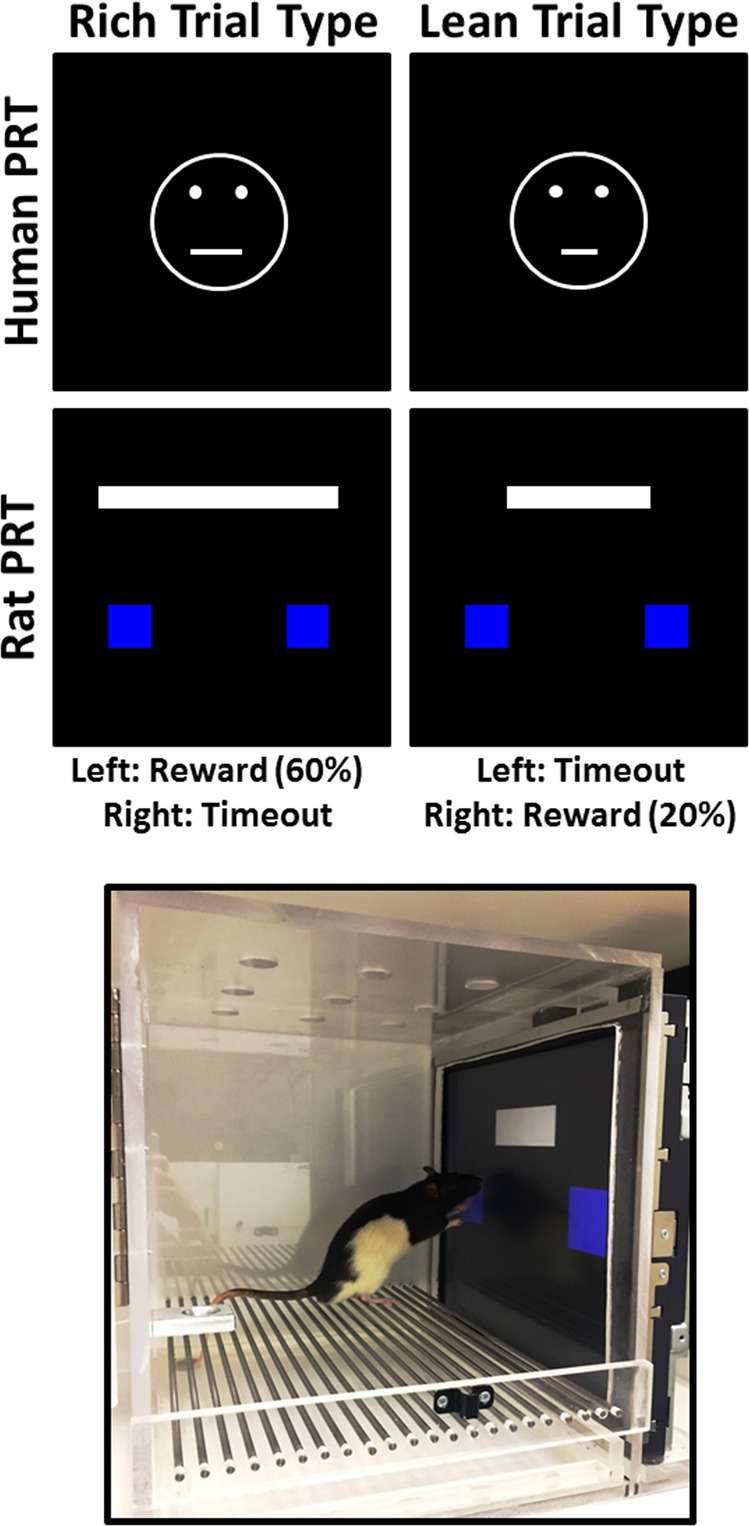
Reverse-translation overview. Task schematic for human PRT (top) and rat PRT (middle) and photograph of rat responding to the short line (bottom).

The goal of the present studies was to address these two limitations with a touchscreen-based PRT, using visual stimuli and line-length discriminations with formal similarity to the human PRT task (see Fig. 1 for analog human/rat task schematics). Two experiments were conducted to examine parametrically key features of the reverse-translated task. In Experiment 1, response biases were assessed with 3:1 (60%:20%) probabilistic contingencies arranged in accord with the human task protocol. In addition, a range of rich:lean probabilities were examined to determine functional relationships between the level of asymmetry between rich and lean probabilities and biased responding. In Experiment 2, PRT performance was examined under 3:1 (60%:20%) probabilistic contingencies using line stimuli that varied in length to determine functional relationships between differences in line length and task discriminability. In Experiment 3, the effects of mechanistically diverse drugs were examined to assess the ability of drugs to modulate response bias. *d*-Amphetamine was studied to evaluate correspondence of drug effects under the present conditions and those previously observed with the auditory rat PRT^33,36^. Scopolamine was evaluated because it has been shown to have clinical efficacy as a fast-acting antidepressant^37,38^ and produces antidepressant-like effects in rodent preclinical models such as the forced swim task^39,40^. Oxycodone was studied to determine how a µ-opioid, which has euphoriant effects but no known antidepressant actions, affects PRT performance. Finally, because diagnoses of mood disorders, including MDD, are more prevalent in female patients^41^, it is important to determine whether this rodent task will yield orderly findings in both sexes for future preclinical drug development efforts. Therefore, in Experiment 4, PRT performance outcomes were examined in female rats to verify their ability to effectively engage in this assay and ascertain whether any sex differences would be revealed using the experimental conditions optimized during Experiments 1–3.

## Methods

### Subjects

Twenty-four adult Long–Evans rats (16 males, 8 females) obtained from Charles River Laboratories (Wilmington, MA), weighing between 250 and 300 g, were housed in a climate-controlled vivarium with a 12-h light/dark cycle (lights on at 7:00 a.m.). Subjects had unrestricted access to water in their home cage and, to establish sweetened condensed milk as a reinforcer, were food restricted via daily post-session portions of ~10–15 g of rodent chow. Experimental sessions were conducted 5 days a week (Monday–Friday). The protocol was approved by the Institutional Animal Care and Use Committee at McLean Hospital and in accordance with guidelines from the Committee on Care and Use of Laboratory Animals of the Institute of Laboratory Animals Resources, Commission on Life Sciences^42^.

### Apparatus

Details and schematics of the rat touch-sensitive experimental chamber can be found here^43^. Briefly, a Plexiglas chamber (25 × 30 × 35 cm^3^) was situated in a sound- and light-attenuating enclosure (40 × 60 × 45 cm^3^). A 17 in touch-sensitive screen (1739L, ELO TouchSystems, Menlo Park, CA) comprised the inside right-hand wall of the chamber. An infusion pump (PHM-100-5, Med Associates, St. Albans, VT) outside the enclosure was used to deliver sweetened condensed milk solution (Sysco Corporation, Houston, TX) into the shallow reservoir (diameter: 3 cm) of a custom-designed aluminum receptacle (4 × 5 × 1 cm^3^) that was mounted 2 cm above the floor bars and centered on the left-hand inside wall. A speaker bar (NQ576AT, Hewlett-Packard, Palo Alto, CA) mounted above the touchscreen was used to emit audible feedback. All experimental events and data collection were programmed in E-Prime Professional 2.0 (Psychology Software Tools, Inc., Sharpsburg, PA).

### Procedure

#### Line-length discrimination training

Modified response-shaping techniques were used to train rats to engage with the touchscreen^44^. A 5 × 5 cm^2^ blue square on a black background served as a response box and was centered on the touchscreen with its lower edge 10 cm above the floor bars. This required the rat to rear on its hind legs to make a touchscreen response with its paw. Each response was reinforced with 0.1 ml of 30% sweetened condensed milk, paired with an 880-ms yellow screen flash and a 440 Hz tone, and followed by a 5-s blackout period. Following reliable responding, the position of the response box was alternated 5 cm left and right of center across 100-trial training sessions. After responses with latencies <5 s were reliably observed to each position, line-length discrimination training commenced. Discrete trials began with presentation of a white line, with its lower edge presented 1.5 cm above the left and right response boxes. The length of the line was either 600 px (31.5 cm: long line) or 200 px (10.5 cm: short line), the width of both lines was 120 px (6.5 cm). Long and short line-length trial types varied in a quasi-random manner across 100-trial sessions such that there were exactly 50 trials of each type, but a given trial type would not be presented >5 times in a row. Subjects were trained to respond to the left or right response box depending on the length of the white line (long line: respond left, short line: respond right, or vice versa). Response box designation was counter-balanced across subjects. A correct response was reinforced as described above and was followed by a 5-s blackout period, whereas an incorrect response immediately resulted in a 10-s blackout period. A correction procedure was implemented during initial discrimination training in which each incorrect trial was repeated until a correct response was made^45^, and was discontinued after <10 repeats of each trial type occurred per session. Discrimination training sessions continued without correction until accuracies for both line-length trial types were ≥80% correct for three consecutive sessions, concordant with the performance criterion of 75–85% correct in previous PRT studies with human participants. Following line-length discrimination training, weekly (Monday–Friday) protocols were arranged such that sessions were conducted on Monday and Tuesday using the line-length stimuli described above in which all correct responses were rewarded [1:1 (100%:100%)]. The line length to be associated with the rich and lean contingency for the remainder of the week was determined for each subject during these two training sessions by examining their accuracies and designating the line length with a higher mean accuracy as the stimulus to be rewarded on the lean schedule. This was designed to examine the effects of procedural variables (Experiments 1 and 2), drug administration (Experiment 3), and sex (Experiment 4) on response bias, rather than the amplification of a preexisting *inherent bias* that is a function of uncontrolled variables.

### Experiment 1: parametric assessment of asymmetry in probabilistic schedules

A group of male rats (*n* = 8) was used to examine the effects of varying the asymmetry of rich:lean probabilities during weekly 5-session conditions (Monday–Friday). Training sessions were conducted each Monday and Tuesday as described above in which all correct responses were rewarded [1:1 (100%:100%)]. During test sessions conducted Wednesday, Thursday, and Friday using probabilistic schedules, subjects were first exposed to a 3:1 (60%:20%) rich:lean probabilistic schedule of reward, in accord with the human task protocol [60% of correct responses to one of the line lengths (rich alternative) and 20% of correct responses to the other line length (lean alternative)]. Incorrect responses were never rewarded. Subsequently, 4:1 (80%:20%), 2:1 (40%:20%), and 1:1 (20%:20%) rich:lean probabilistic contingencies were examined, in that order, in successive weeks.

### Experiment 2: parametric assessment of disparity in line-length stimuli

A separate group of male rats (*n* = 8) was used to study the effects of varying the disparity in long and short line lengths during weekly 5-session conditions (Monday–Friday). Training sessions were conducted each Monday and Tuesday as described above in which all correct responses were rewarded [1:1 (100%:100%)]. Using the 3:1 (60%:20%) rich:lean probabilistic schedule described in Experiment 1, subjects were exposed to the same long and short line lengths for 3 daily sessions conducted Wednesday, Thursday, and Friday. Subjects were first exposed to the line-length stimuli used in Experiment 1, that is, long and short line varied by 400 px (600:200 px). Subsequently, the difference between long and short line lengths was reduced weekly. Long and short line-length differences of 200 px (500:300 px), 100 px (450:350 px), 50 px (425:375 px), and 25 px (413:388 px) were examined, in that order, each week.

### Experiment 3: effects of drug treatment on PRT performance

Studies of how selected drugs modified PRT performance were conducted by exposing the male rats from Experiment 1 (*n* = 8) to a weekly (Monday–Friday) acute drug testing protocol as follows: training sessions on Monday and Tuesday were conducted using the 600:200 px long and short lines in which all correct responses were rewarded [1:1 (100%:100%)]. The 3:1 (60%:20%) rich:lean probabilistic contingencies were used for the remainder of the week, and the effects of drug administration on PRT performance were examined in the fifth (Friday) session. Saline or doses of *d*-amphetamine (0.32–3.2 mg/kg), scopolamine (0.1–1.0 mg/kg), or oxycodone (0.1–1.0 mg/kg) were administered 15 min prior to the session. As in Experiments 1 and 2, during drug testing sessions, there were no response omission criteria (i.e., limited hold) that advanced trials if no response occurred. However, if a subject failed to respond on the touchscreen within 30 min post-drug administration, the session was terminated. Drugs were studied using a within-subject design in which all rats were tested with saline and all doses of each drug in a mixed order across subjects.

### Experiment 4: effects of sex on PRT performance

A group of female rats (*n* = 8) was used to determine whether any sex differences would be observed in PRT performance outcomes using the task parameters optimized in Experiments 1–3 (i.e., 60%:20% rich:lean probabilities and 600:200 px long and short lines). Following line-length discrimination training as described above, a 1-week test was comprised of training sessions conducted on Monday and Tuesday using the 600:200 px long and short lines in which all correct responses were rewarded [1:1 (100%:100%)]. During test sessions conducted on Wednesday, Thursday, and Friday using probabilistic schedules, subjects were exposed to a 3:1 (60%:20%) rich:lean probabilistic schedule of reward in accord with Experiments 2 and 3, and the human task protocol.

### Data analysis

The implementation of probabilistic contingencies yields two primary dependent measures: response bias and task discriminability, which can be quantified using equations derived from signal detection theory by examining the number of _Correct_ and _Incorrect_ responses for Rich and Lean trial types. Response bias is calculated using the following log *b* equation:

1 $$\log\,b = 0.5 \times {\log} \left( {\frac{{\left( {{\mathrm{Rich}}_{\mathrm{Correct}} + 0.5} \right) \times \left( {{\mathrm{Lean}}_{\mathrm{Incorrect}} + 0.5} \right)}}{{\left( {{\mathrm{Rich}}_{\mathrm{Incorrect}} + 0.5} \right) \times \left( {{\mathrm{Lean}}_{\mathrm{Correct}} + 0.5} \right)}}} \right).$$

High bias values are produced by high numbers of correct responses for rich trials and incorrect responses for lean trials. Discriminability is calculated using the following log *d* equation:

2 $$\log\,d = 0.5 \times {\log} \left( {\frac{{\left( {{\mathrm{Rich}}_{\mathrm{Correct}} + 0.5} \right) \times \left( {{\mathrm{Lean}}_{\mathrm{Correct}} + 0.5} \right)}}{{\left( {{\mathrm{Rich}}_{\mathrm{Incorrect}} + 0.5} \right) \times \left( {{\mathrm{Lean}}_{\mathrm{Incorrect}} + 0.5} \right)}}} \right).$$

High discriminability values are produced by high numbers of correct responses for both rich and lean trials. (0.5 is added to all parameters in both Eqs. (1) and (2) to avoid instances where no errors are made on a given trial type, thus making log transforms impossible.) The utility of these equations has been repeatedly confirmed in prior human studies^26–30,34^ and rodent studies^33,36^. In addition, accuracy (percent correct) and reaction time (latency from line presentation to response) were calculated and presented as session-wide group means (±SEM) for rich and lean trials. All data (log *b*, log *d*, accuracy, reaction time) were subject to repeated-measures analysis of variance (ANOVA). For accuracy and reaction time, the repeated-measures factor trial type (rich vs. lean) was added to the model. When appropriate, ANOVAs were followed by post hoc tests for linear trends to evaluate the statistical significance of increasing asymmetry of rich:lean probabilities and disparity of long and short line length, and Bonferroni’s tests to evaluate the statistical significance of rich and lean trial type on accuracy and reaction time, as well as the statistical significance of drug doses compared to saline treatment. Paired *t* tests were used to evaluate possible sex differences in log *b*, log *d*, accuracy, and reaction time. The criterion for significance was set at *p* < 0.05. All statistical analyses were conducted using GraphPad Prism 5 Software (San Diego, CA, USA).

### Drugs

*d*-Amphetamine (dextroamphetamine hemisulfate), scopolamine (scopolamine hydrobromide), and oxycodone (oxycodone hydrochloride) were obtained from Sigma-Aldrich (St. Louis, MO). All drugs were dissolved in 0.9% saline solution and were administered via subcutaneous injection in volumes of 1 ml/kg or less. Drug doses (mg/kg) are expressed in terms of their free base weights.

## Results

### Experiment 1: parametric assessment of asymmetry in probabilistic schedules

**Fig. 2:**
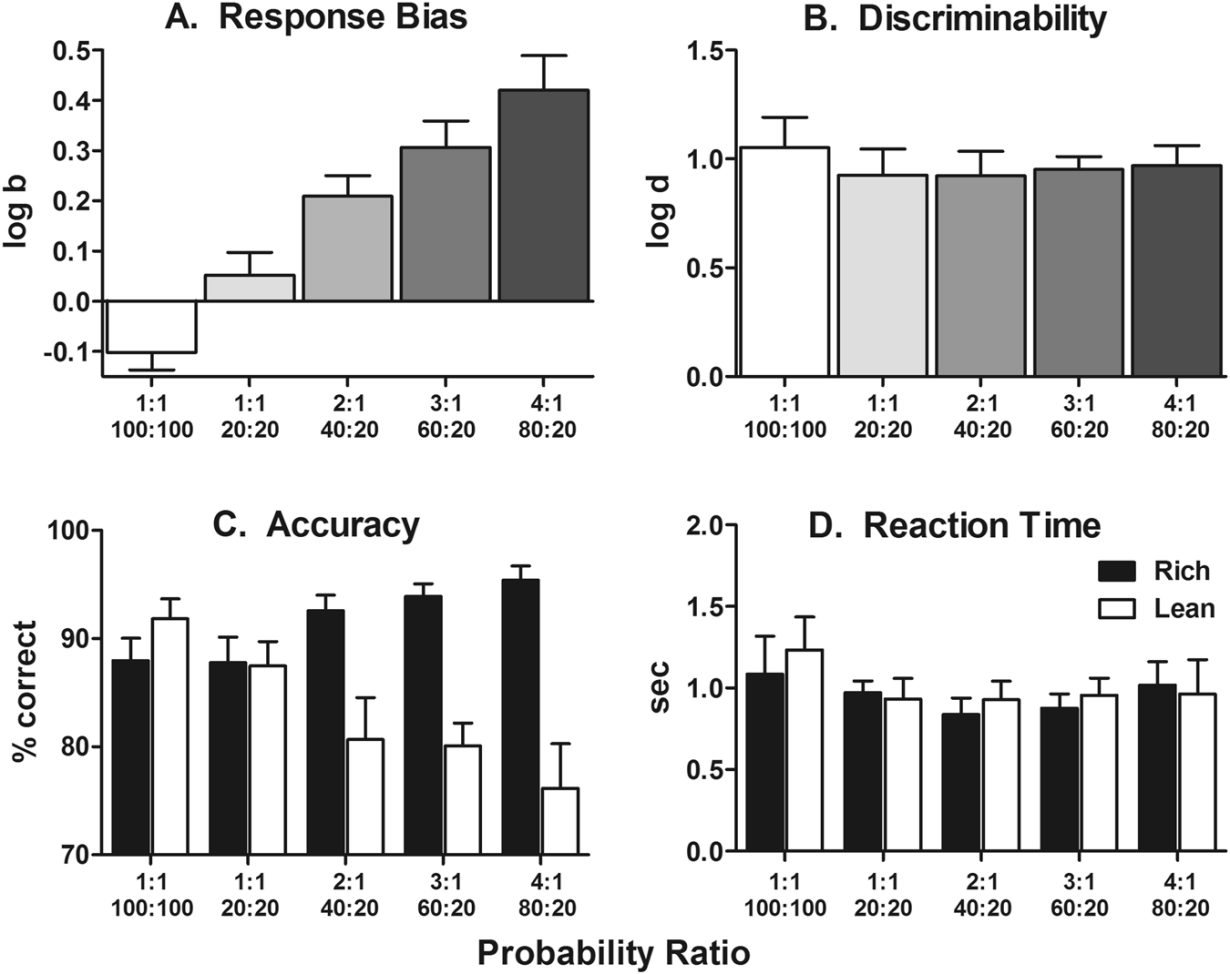
Parametric assessment of asymmetry in probabilistic schedules. Group mean (±SEM) log*b* (top-left), log*d* (top-right), accuracy (bottom-left), and reaction time (bottom-right) as a function of rich:lean probabilistic contingencies. *n* = 8.

All subjects learned the line-length discrimination to ≥80% correct criterion following, on average, 15.1 (range: 9–25) sessions of training. Figure 2 presents findings from the parametric assessment of rich:lean probabilistic contingencies. As expected, prior to the introduction of probabilistic contingencies, a small inherent bias to either the long or short line was observed and varied across subjects. The line length that was associated with inherent bias was designated as the lean trial type and, as such, 1:1 (100%:100%) ratios are represented in Fig. 2 with relatively small negative log *b* values (Fig. 2a). Exposure to 2:1 (40%:20%), 3:1 (60%:20%), and 4:1 (80%:20%) probabilistic contingencies produced a response bias (log *b*) that corresponded to the asymmetry of rich:lean probabilities (Fig. 2a). That is, larger differences between rich and lean probabilities produced larger log *b* values. Exposure to 1:1 (20%:20%) contingencies was associated with a near-zero group average log *b* and re-emergence of the small inherent bias observed prior to exposure to the probabilistic conditions in two of eight subjects. The relationship between increases in asymmetry of rich:lean probabilities and increases in log *b* values was orderly and significant [*F*(4,28) = 17.92, *p* < 0.0001], as was the linear trend (*p* < 0.0001). As shown in Fig. 2b, changes in response bias across all conditions tested were produced without changes in task discriminability (log *d*), which remained relatively constant across all probabilistic contingencies [*F*(4,28) = 0.40, *p* = 0.81]. The manner in which response biases increased without changes in task discriminability is reflected in systematic changes in accuracies (percent correct) under the rich:lean trial types (Fig. 2c). That is, increasing the asymmetry between rich and lean probabilities of reward significantly increased accuracy of rich trial types [*F*(4,28) = 5.41, *p* = 0.002] with a linear trend (*p* < 0.0001), while concurrently decreasing accuracy of lean trial types significantly [*F*(4,28) = 5.68, *p* = 0.002] with a linear trend (*p* < 0.0001). In addition, there was a significant interaction between probabilistic condition and trial type [*F*(4,56) = 15.17, *p* = 0.002]. Figure 2d depicts reaction time which was, on average, ~1 s with no significant differences observed across probabilistic conditions [*F*(4,56) = 1.07, *p* = 0.38].

### Experiment 2: parametric assessment of disparity in line-length stimuli

**Fig. 3:**
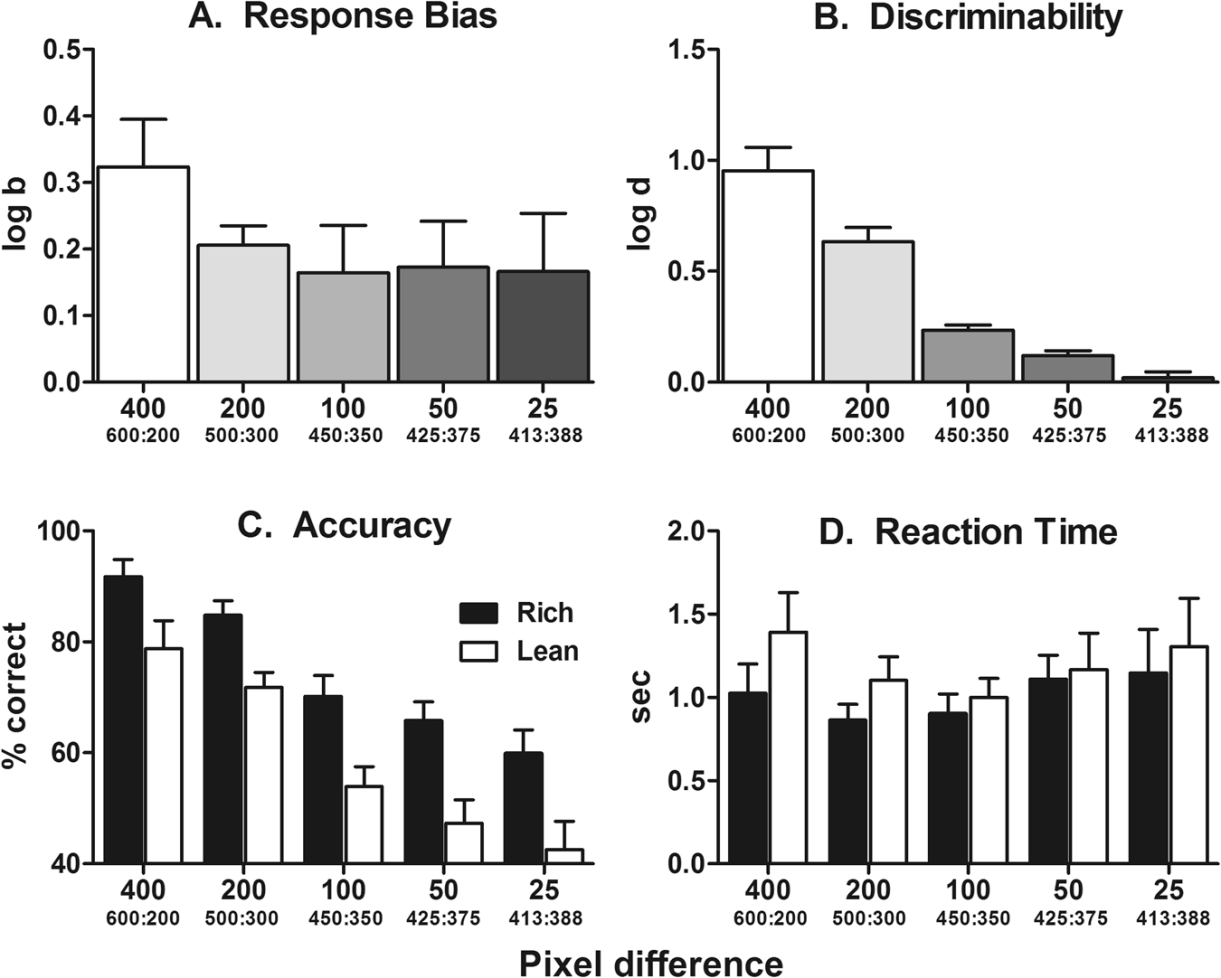
Parametric assessment of disparity in line-length stimuli. Group mean (±SEM) log*b* (top-left), log*d* (top-right), accuracy (bottom-left), and reaction time (bottom-right) as a function of long:short line-length stimuli. *n* = 8.

All subjects learned the line-length discrimination to ≥80% correct criterion following, on average, 18.1 (range: 13–23) training sessions. Figure 3 presents findings from the parametric assessments of reducing discriminability by reducing the difference between long and short line lengths. As shown in Fig. 3b, the relationship between decreases in the difference between long and short line lengths and decreases in log *d* values was orderly and significant [*F*(4,28) = 58.90, *p* < 0.0001] as was the linear trend (*p* < 0.0001). As illustrated in Fig. 3a, although log *b* values were reduced by decreasing the difference between long and short line lengths from 400 px (600:200 px) to 200 px (500:300 px), there were no significant differences in log *b* across the line lengths tested [*F*(4,28) = 1.23, *p* = 0.32]. Accuracies were significantly reduced during both rich [*F*(4,28) = 19.42, *p* < 0.0001] and lean [*F*(4,28) = 16.56, *p* < 0.0001] trial types as the differences in line lengths decreased (Fig. 3c). The absolute difference in percent correct (accuracy) between rich and lean trial types remained relatively constant across line-length conditions, ranging on average from 13 to 18% across all line lengths tested. Moreover, reducing the difference between long and short line lengths significantly increased the reaction time across line-length conditions [*F*(4,56) = 2.95, *p* = 0.03] (Fig. 3d). Mean reaction times were, on average, always slightly (<0.5 s) longer during lean trial types, regardless of line-length condition; however, interaction between trial types was not statistically significant [*F*(1,14) = 0.62, *p* = 0.45].

### Experiment 3: effects of drug treatment on PRT performance

**Fig. 4:**
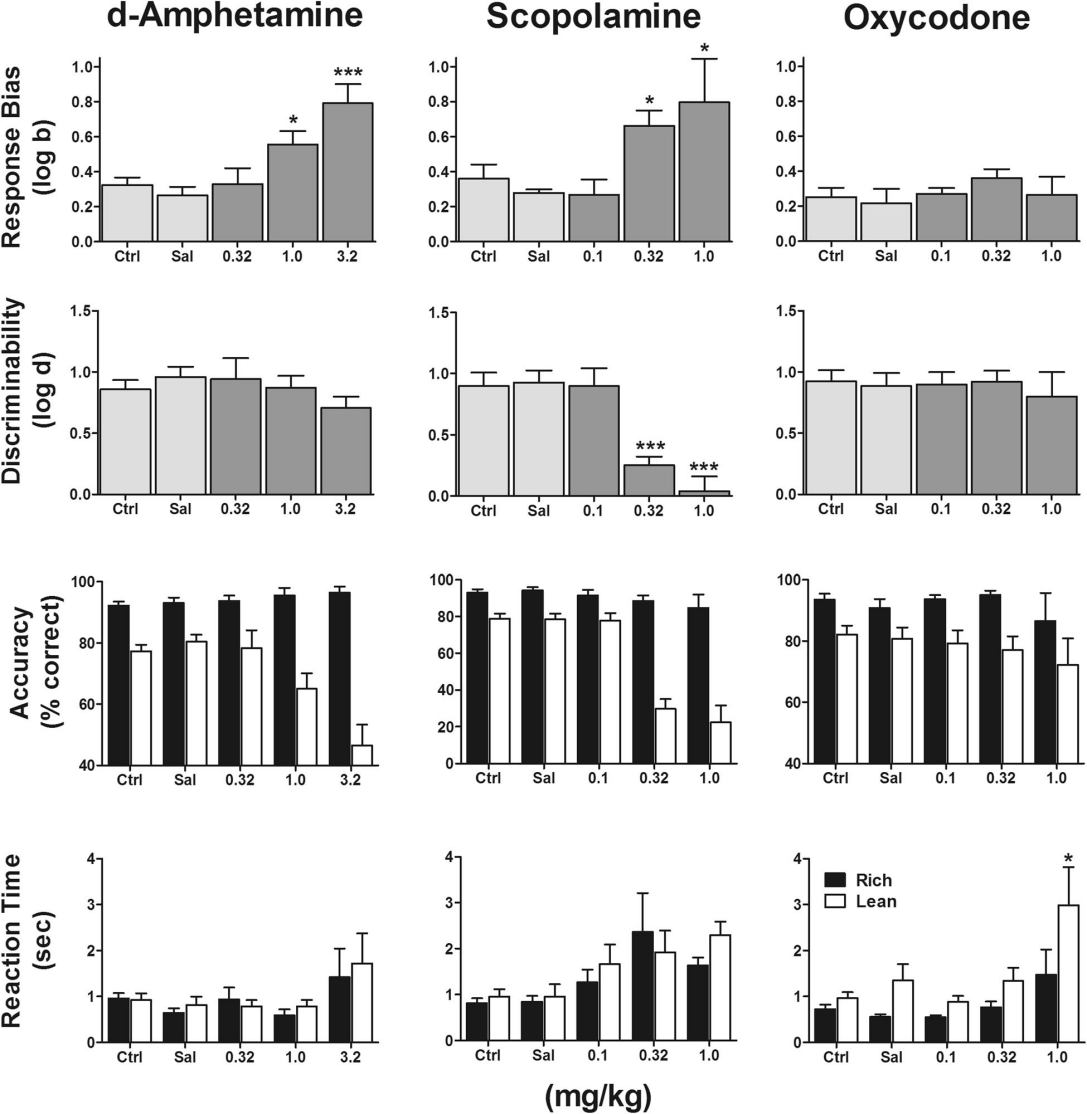
Effects of drug treatment on PRT performance. Group mean (±SEM) log*b* (first row), log*d* (second row), accuracy (third row), and reaction time (fourth row) during control (Ctrl) sessions that were conducted the day before sessions in which saline (Sal) or doses of *d*-amphetamine (left column), scopolamine (middle column), and oxycodone (right column) were administered. *p < 0.05; ***p < 0.001. *n* = 7, 0.32 mg/kg scopolamine; *n* = 5, 1.0 mg/kg scopolamine; *n* = 7, 1.0 mg/kg oxycodone; otherwise *n* = 8.

Figure 4 presents the effects of several drugs on measures of PRT performance.

#### *d*-Amphetamine

The monoaminergic indirect agonist *d*-amphetamine produced significant dose-related increases in log *b* [*F*(3,21) = 12.00, *p* < 0.0001] following administration of 1.0 mg/kg (*p* < 0.05) and 3.2 mg/kg (*p* < 0.0001). These *d*-amphetamine-mediated increases in response bias were observed without changes in log *d* [*F*(3,21) = 0.92, *p* =0.45], owing to dose-dependent (albeit nonsignificant) increases in accuracy during the rich trial type that were small [*F*(3,21) = 0.85, *p* =0 .48] and significant dose-dependent decreases in accuracy during the lean trial type [*F*(3,21) = 7.66, *p* = 0.001]. Reaction time was increased following 3.2 mg/kg; however, these effects lay just outside statistical significance [*F*(3,42) = 2.78, *p* = 0.053]. The higher dose of 10 mg/kg *d*-amphetamine produced untoward behavioral effects in the first subject, precluding further assessment in additional subjects.

#### Scopolamine

The muscarinic antagonist scopolamine also significantly increased log *b* [*F*(3,24) = 5.56, *p* = 0.005] following administration of 0.32 (*p* < 0.05) and 1.0 mg/kg (*p* < 0.05). However, these doses of scopolamine also produced significant (0.32 mg/kg: *p* < 0.001; 1.0 mg/kg: *p* < 0.001) decreases in log *d* [*F*(3,24) = 14.30, *p* < 0.0001]. These effects on log *d* reflect significant decreases in accuracy during the lean trial type [*F*(3,24) = 33.98, *p* < 0.0001] and small but dose-dependent decreases in accuracy during the rich trial type [*F*(3,24) = 1.18, *p* = 0.34]. Moreover, administration of 0.32 and 1.0 mg/kg scopolamine abolished responding in, respectively, one and three of the eight subjects. For the subjects that did respond following scopolamine administration, significant dose-dependent increases in reaction time were observed [*F*(3,48) = 3.48, *p* **=** 0.02], with no interaction between rich and lean trial types [*F*(1,48) = 0.34, *p* = 0.56].

#### Oxycodone

The µ-opioid agonist oxycodone failed to significantly alter either log *b* [*F*(3,27) = 0.74, *p* = 0.54] or log *d* [*F*(3,27) = 0.17, *p* = 0.92] across all doses tested. In addition, accuracy was unchanged during both rich [*F*(3,27) = 0.67, *p* = 0.58] and lean [*F*(3,27) = 0.45, *p* = 0.72] trial types. Administration of 1.0 mg/kg oxycodone abolished responding in one subject and significantly increased mean reaction time in the remaining 7 subjects [*F*(3,54) = 6.45, *p* = 0.0008], with significant interaction between rich and lean trial types [*F*(1,54) = 9.67, *p* = 0.003].

### Experiment 4: effects of sex on PRT performance

**Fig. 5:**
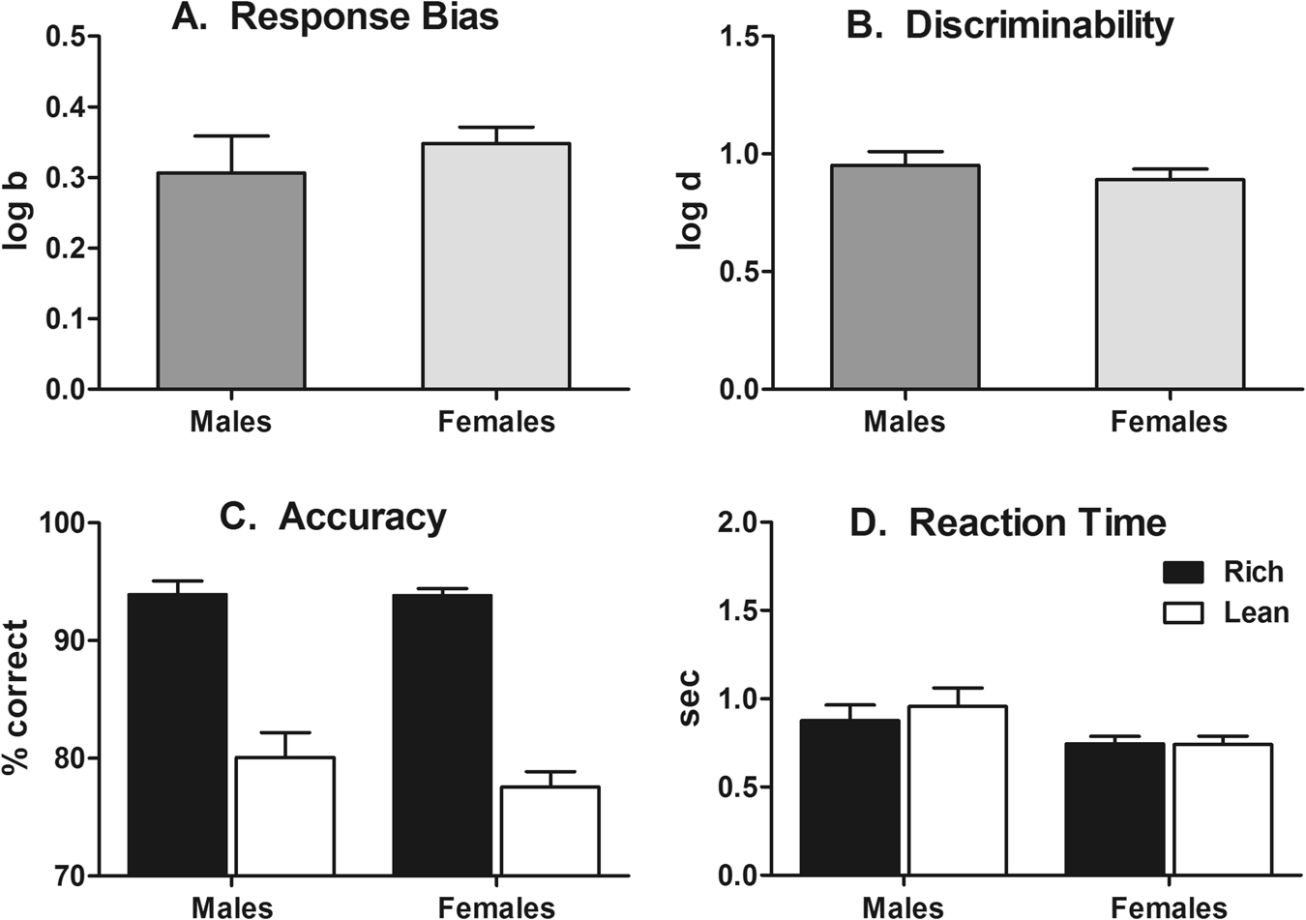
Effects of sex on PRT performance. Group mean (±SEM) log *b* (top-left), log *d* (top-right), accuracy (bottom-left), and reaction time (bottom-right) observed in male (*n* = 8) and female (*n* = 8) rats.

All subjects learned the line-length discrimination to ≥80% correct criterion following, on average, 15.9 (range 10–24) training sessions. Figure 5 presents PRT performance outcomes in female rats. To aid evaluation of sex differences, data from male rats under identical task parameters and contingencies (i.e., 60%:20% rich:lean probabilities and 600:200 px long and short lines) from Fig. 2 are also plotted in Fig. 5 to juxtapose with data from female rats. As evident across all PRT performance measures, highly similar outcomes and no statistical differences between sexes were observed in log *b* [*t*(14) = 0.80, *p* = 0.45], log *d* [*t*(14) = 1.48, *p* = 0.18], rich [*t*(14) = 0.07, *p* = 0.95] or lean [*t*(14) = 1.71, *p* = 0.13] accuracies, and rich [*t*(14) = 1.74, *p* = 0.13] or lean [*t*(14) = 1.73, *p* = 0.13] reaction time measures.

## Discussion

The present studies were conducted to empirically validate a touchscreen-based PRT task to examine reward learning in rats. Parametric assessments of rich:lean probabilities verified that changes in response bias (log *b*) correlated positively with the asymmetry between rich and lean probabilities. Parametric assessments of difference in line-length stimuli also verified that changes in task discriminability (log *d*) correlated positively with differences between long and short line length. Importantly, the contingencies and stimuli optimized for subsequent drug testing (i.e., 60%:20%, rich:lean; 400:200 px, long:short) produced log *b* values (~0.2–0.3) and accuracies (~75–85% correct) that are highly similar to those in previous studies with healthy control human participants^19,25,34^. Collectively, the present studies optimized and validated a touchscreen-based PRT in rats that is both formally and functionally similar to the prototypical computerized version used with humans in clinical settings. This equivalence is translationally advantageous for parallel studies of reward responsiveness across rats and human participants. Our studies also indicate that PRT performance criteria can be achieved in a relatively short time (mean: 16.6 training sessions [range 9–25; *n* = 16]), which is a >50% reduction in training time reported previously with rats using the auditory variant (~40 training sessions^33,36^).

Pharmacological manipulations verified selectivity of drug action in this task. For example, as demonstrated previously^33,36^ and replicated here, *d*-amphetamine increased log *b* without altering log *d* in a dose-related manner. These results presumably reflect *d*-amphetamine-induced increases in reward responsiveness via increased striatal dopamine transmission in the rat^46^, a mechanism that has been hypothesized to modulate reward learning in humans^34,47,48^. Indeed, these effects are consistent with converging evidence that dopamine plays a pivotal role in reward learning^49^, whereas dysfunction in mesolimbic dopamine transmission can be associated with anhedonic phenotypes^50^. Moreover, the pro-hedonic actions of *d*-amphetamine are proposed to play a role in its reported antidepressant effects^51^, although well-controlled clinical investigations are needed to confirm this. Scopolamine also produced significant increases in response bias; however, these increases were accompanied by decreases in task discriminability. The latter effects are likely a function of scopolamine’s anti-muscarinic actions and confirm cognition-disruptive effects that have been observed previously in rodents^52,53^. Although scopolamine has shown some promise as a fast-acting antidepressant^37,38^, its ability to increase reward learning is currently unclear and, as shown in the PRT, remains indeterminate due to its adverse effects on task performance. Finally, oxycodone failed to produce changes in either response bias or task discriminability across a range of doses. These findings following oxycodone treatment provide informative negative control information, showing that drugs with well-known euphoriant effects but without known antidepressant effects, such as µ-opioid agonists, will not produce positive effects in this assay of reward learning.

The greater prevalence of mood disorder diagnoses in female patients^41^ necessitates study of both sexes during preclinical drug development. Although male rats were used in the parametric and pharmacological experiments to empirically validate the rodent PRT task, subsequent studies with female rats under conditions optimized in Experiments 1–3 yielded highly similar performance outcomes across all dependent measures (Fig. 5). We cannot presently be certain whether sex differences will emerge following drug treatment. However, it is important, although not surprising, that performance in female rats showed similar sensitivity to probabilistic conditions, yielding response biases and task discriminability that closely approximated findings in male rats. This is consistent with findings in humans across multiple studies, which have not yielded sex differences in any of the PRT variables^26–30,34^.

Finally, it is encouraging that, even in the absence of any programmed stressors to decrease reward responsiveness, response bias could be enhanced by drug treatment. The extent to which reward learning is blunted following exposure to programmed stress conditions (e.g., social defeat, inescapable electric shock, chronic mild stress) can subsequently be rescued following drug treatment will be an important complementary approach in future research with the touchscreen-based rodent PRT. Of note, although the present findings provide compelling evidence for the translational potential of this assay, it is important to emphasize that, as discussed earlier, response bias in the PRT does not capture the full spectrum of anhedonia, which is itself heterogeneous. Thus, PRT performance cannot be equated to *anhedonia* per se. Rather, we believe that PRT performance (specifically, response bias) provides a reliable and objective measure of reward learning, that is, the ability to modulate behavior as a function of reward, which is an important RDoC Positive Valence Systems subdomain implicated in anhedonic phenotypes. Other assays—including the Effort Expenditure for Rewards Task^54^, which has been modeled after analogous tasks in rodents^55^—will be needed to assess the translational impact of other reward subdomains. Cognizant of this consideration, the present results provide key data suggesting that this rodent variant of the PRT may be applicable toward the discovery of novel therapeutics for conditions in which deficits in reward learning is a cardinal phenotype.
